# Powassan Virus—A New Reemerging Tick-Borne Disease

**DOI:** 10.3389/fpubh.2017.00342

**Published:** 2017-12-12

**Authors:** Syed Soheb Fatmi, Rija Zehra, David O. Carpenter

**Affiliations:** ^1^Institute for Health and the Environment, University at Albany, SUNY, Rensselaer, NY, United States

**Keywords:** ticks, *Ixodes*, *scapularis*, Powassan virus, vector-borne diseases, deer-tick virus, flavivirus, meningoencephalitis

## Abstract

Powassan virus is a neurovirulent flavivirus consisting of two lineages causing meningoencephalitis. It is the only member of the tick-borne encephalitis serogroup which is present in mainland North America. With a total number of 27 cases from 1958 to 1998 and 98 cases from 1999 to 2016, reported cases have increased by 671% over the last 18 years. Powassan infection is transmitted by different tick species in different geographical regions. *Ixodes scapularis* is the primary vector that transmits the virus on the East Coast of US and *Ixodes cookei* in the Midwest and Canada, while *Hemaphysalis longicornis* is the vector in Russia. Powassan has no singular pathognomonic finding and presents with a wide spectrum of symptoms including severe neurological symptoms. The clinical challenge lies within the management of the disease as there is no standard diagnostic protocol and most cases are only diagnosed after a patient goes through an extensive workup for other infectious disease. The diagnosis is established by a combination of imaging and serologic tests. In case of Powassan meningoencephalitis, computed tomography scan and magnetic resonance imaging show vascular insults, which are also seen in cases of tick-borne encephalitis virus, another flavivirus of medical importance. Serologic tests are the gold standard for diagnosis, although testing is not widely available and only state health departments and Center for Disease Control and Prevention can perform Powassan-specific IgM antibody testing utilizing enzyme-linked immunosorbent assay and immunofluorescence antibody. Powassan is also of veterinary medical importance. Wildlife animals act as a reservoir to the pathogens, hence possessing threat to humans and domestic animals. This review highlights Powassan’s neurotropic presentation, epidemiology, diagnostic challenges, and prevalence. Strong emphasis is placed on establishing diagnostic protocols, widespread Powassan-specific IgM testing, role of the vector in disease presentation, and necessary preventive research.

## Introduction

Powassan virus (POWV) is a fatal, neurotropic “tibovirus” (1), and is the only member of tick-borne encephalitis serogroup present in North America (2). With a reported 671% rise in reported cases in the last 18 years as compared to the previous 40 years, POWV has become an emerging danger worldwide (3, 4). Powassan infection in humans has been rare and comparative prevalence is less than other diseases. The prevalence of POWV in endemic areas may be much higher than originally believed. Some asymptomatic people in these areas have serologic evidence of the virus while getting tested for other infectious disease. Others develop moderate to fatal symptoms. The fatality rate is 10%, and about 50% of those people who develop neurological symptoms end up with long-term sequelae (2).

Tick-borne diseases are a major public health concern from the Great Lakes region of the mid-eastern United States to the sub-Saharan desserts of Africa. Ticks carry agents that cause a wide array of clinical pathologies which are protozoal, bacterial, or viral in origin. POWV was first reported in 1958 in Powassan, Ontario (5). Powassan is endemic in the northeast and upper midwest of the United States, and cases have also been reported in Far-Eastern Russia (6). Like other tick-borne diseases, Powassan is a diagnostic challenge because of its versatility of clinical presentation and the unpredictability of the course of the illness. Unfortunately, there is a lack of diagnostic methods that complement the severity and aggressiveness of the disease and its complex genetic structure.

There are more than 15 infectious diseases that are transmitted by ticks (7). The most well-known is Lyme disease, caused by a bacterium. With the large number of cases, pathognomonic findings, and insidious onset of symptoms, Lyme is readily being identified and treated. Powassan, on the other hand, poses a greater uncertainty due to its rapid onset of neuro-invasive symptoms, the brief transmission time, and less reliable serologic testing. While tick-borne encephalitis virus (TBEV) is commonly seen across Europe, Powassan is the only tick-borne flavivirus that is endemic in the western hemisphere (8). Powassan is one of the least studied flavivirus and there is an urgent need for further exploration and in-depth research into this neurotropic virus.

With a significant increase in the number of Powassan cases in the last decade, there is an urgent need for further research and understanding of the virus, the vector, and the disease. There have been number of reviews on POWV in the past; however, most have either dealt only with case presentations, genetic makeup, or *Flaviviruses* in general. This review encompasses all factors related to POWV, including the taxonomy and lifecycle of the vector, genetic makeup, inoculation, and presentation of the virus.

It is critical to highlight the lack of established diagnostic and treatment protocols with the scarcity of available serologic testing for Powassan. This review also emphasizes prevention methods and the direction of future research that is required to effectively manage this reemerging, often fatal, and neuro-invasive disease.

## Method

This review is a literature-based study from previous peer-reviewed articles from PubMed, google scholar, and science direct. It reviews studies done on POWV from the first known literature from 1962 to more recent studies. It is a review on POWV and its comparison to other vector-borne diseases. The data were gathered on epidemiology, etiology, pathophysiology, as well as the clinical and diagnostic understanding from previously reviewed articles, research studies, and medicine textbook. This information was gathered and searched with the use of following keywords: ticks, *Ixodes, scapularis*, POWV, vector-borne diseases, deer-tick virus (DTV), *flavivirus* and meningoencephalitis. Numerical data were gathered from US Environmental Protection Agency, Center for Disease Control and Prevention, and US Geological Survey. Data were analyzed for statistics on recent weather changes, incidence and prevalence, and geographical expansion of the disease.

## The Vector and the Virus

### The Vector

**Figure 1:**
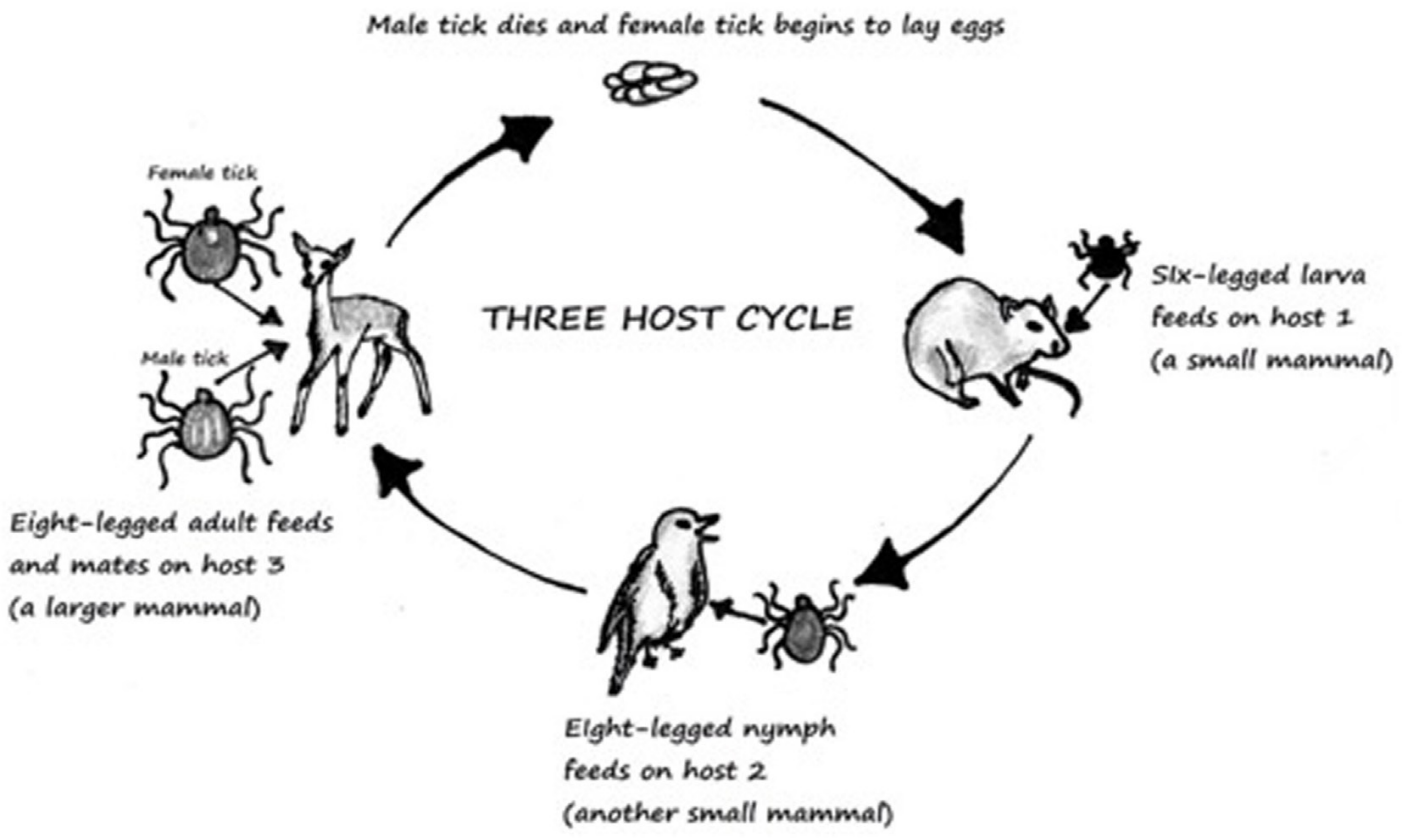
Three-host life cycle of Ixodes. Derived from Ref. (13).

**Figure 2:**
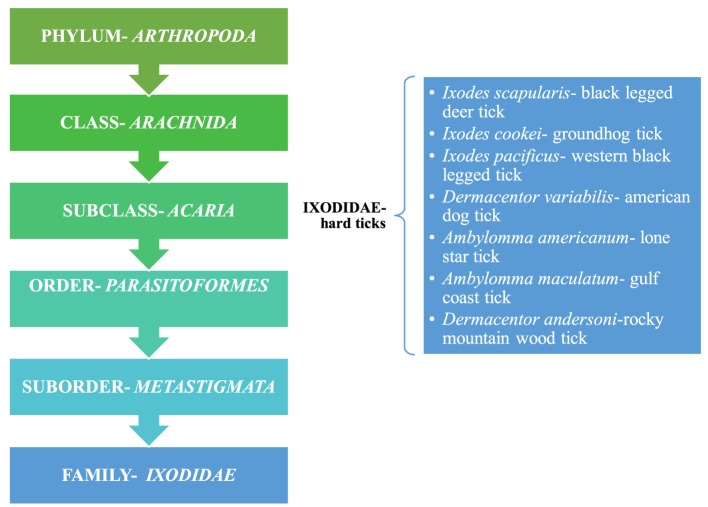
Taxonomical representation of Ixodidae and a listing of hard ticks; Ref. (9).

Powassan is primarily transmitted through ticks, which are pathogen carrying, small blood sucking arthropods that infect humans by biting them. Ticks belong to the class *Arachnida*, order *Parasitoformes*, and suborder *Metastigmata*, which further subdivides into three families; *Ixodidae* (hard ticks), *Argasidae* (soft ticks), and *Nuttalliellidae* (9) (Figure 2). Ticks originated during the “pre-mid Cretaceous period,” with *Ixodids* probably sprouting from the parasites of reptiles during the Paleozoic and Mesozoic era (10). *Ixodidae* is the largest family comprising of 14 genera, which has around 702 species and holds great importance as it relates to infectious diseases seen around the world (11). The lifecycle of *Ixodidae* ticks are somewhat complicated. Some hard ticks stay with the same host throughout their life cycle while others change hosts with each phase of their development (12) (Figure 1). Host selection for the ticks relies upon the physiological characteristics of the host including smell of the body and breath and its thermoregulation (12).

Ticks are known to wait in paths that have been established for host movement in pattern called “questing” and during that time ticks place themselves in the optimal positon for host attachment (12). They must consume blood from the host to survive, and if the host is infected the tick will become infected in the process. This then leads to infection of other hosts. *Ixodid* ticks can rely upon one-, two-, or three-hosts to complete their lifecycle (13). The most common tick causing Lyme and Powassan disease goes through a three-host lifecycle (Figure 1). The full cycle lasts from 2 to 3 years, although most ticks do not end up surviving the full cycle (12, 13). The metamorphosis from one stage to another is dependent upon seasons (13). The host species for each stage is different (13). Usually the first host during the larvae stage is a small rodent which is followed by another rodent, lagomorphs, or a bird in the nymph stage. Final stage is characterized by an adult hosting on a “larger herbivore, carnivore, or human” (13).

*Ixodidae* (hard ticks) are found around the globe and cause a number of diseases, including Crimean-Congo hemorrhagic fever in Europe, China, Africa and Middle East, Kyasanur forest disease in southern India, Omsk hemorrhagic fever in west Siberia, and tick-borne encephalitis in Japan, Russia and Albania (10). In the US, most tick-borne diseases are caused by *Ixodes scapularis, Ixodes cookei, Ambylomma americanum*, and *Dermacentor variabilis* (Table 1). Diseases like Powassan, Lyme, anaplasmosis, and babesiosis are all transmitted by *I. scapularis* also known as the “deer tick” or *Ixodes dammini* (14), which is present in the Northeast, Midwest, and the Gulf coast regions of the US. Even though *I. scapularis* has a wide geographic distribution, there are other non-geographic factors contributing to the higher incidence of tick-borne illnesses in the Northeast and Midwest (14). *I. cookei* is the second vector responsible for transmission of the POWV (15). *I. cookei*, which is more commonly known as “woodchuck or ground hog” tick is quite similar to deer ticks. They are not very common in the tick family worldwide, although they are the second most common ticks from the *Ixodae* family in Maine, USA (15). These ticks are mostly found in the New England, northern Midwest and southern Canada (16). As these areas are cooler, *I. cookei* are seen primarily in the summer (16). Another species of tick, *Hemaphysalis longicornis* is known to transmit the virus in East Russia (17).

**Table 1:** Most common tick-borne diseases in US; Ref. (7).

Diseases	Ticks
Powassan disease	*Ixodes scapularis* and *Ixodes cookei*
Babesiosis	*I. scapularis*
Lyme disease	*I. xodes scapularis* and *Ixodes pacificus*
Rocky mountain spotted fever	*Rhipicephalus sangunineus* and *Dermacentor variabilis*
Stari	*A. americanum*
Ehrlichiosis	*A. americanum*
Anaplasmosis	*I. pacificus*
Tularemia	*D. variabilis*
*Rickettsia parkeri* rickettsiosis	*Ambylomma maculatum*

### The Virus

**Figure 3:**
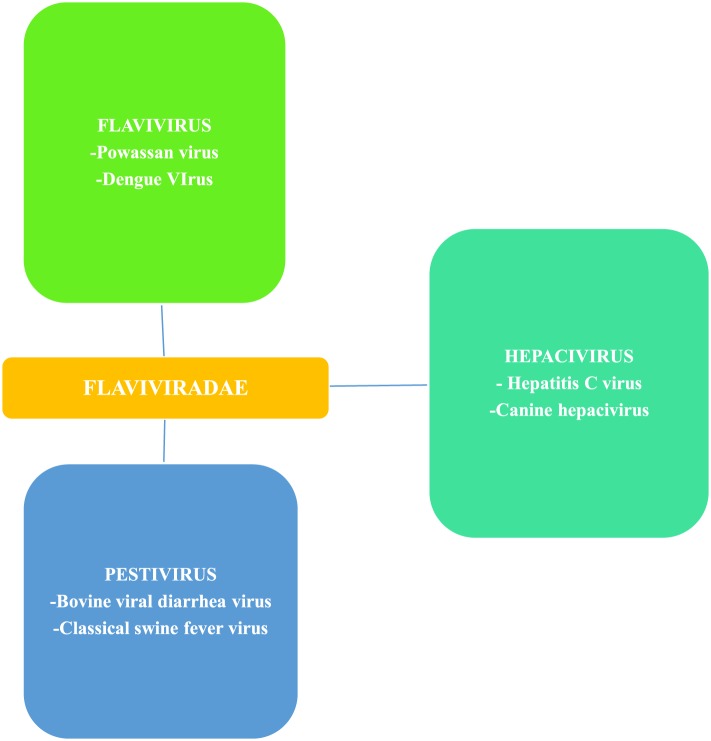
Flaviviradae genus and species overview; Ref. (19).

*Flaviviruses*, of which Powassan is one, are scattered throughout most of the globe and billions of people are at risk of infections (18). *Flavivirus* is a member of the family *Flaviviridae*, which has two other members, *Pestivirus* and *Hepacivirus* (19) (Figure 3). *Flavivirus* gets its name from yellow fever virus, as *Flavus* in Latin stands for yellow (20), echoing the hyperbilirubinemia caused from the hemorrhagic presentation of yellow fever. Similarly, the other two members of Flaviviridae also derive their names from Latin; *Pestis* meaning plague and *Hepatos* meaning liver (19). Members of the *flaviviradae* share morphological and genetic structure as they are all positive sense RNA viruses; however, they still represent unique physiological characteristics and do not always exhibit the same antigenic properties (19).

RNA viruses that are positive sense are classified based on the similarities of their RNA-dependent RNA polymerase (19), which is a key component of their transcription machinery. *Flavivirus* are primarily transmitted through two vectors including ticks and mosquitos. There are more than 70 virus species in the genus (19). Those of medical importance include, POWV, dengue fever, yellow fever, tick-borne encephalitis, and zika virus (21). Powassan and zika have received recent attention because of their geographic spread and the seriousness of the illnesses. Yellow fever and dengue represent viruses capable of causing hemorrhagic sequels. Dengue has a much higher incidence than Powassan or zika with an estimated 50–100 million cases yearly worldwide (22). Tick-borne encephalitis has also been a leading public health concern in Europe and Asia (2).

As a positive sense single strand RNA virus, *Flavivirus* replicates in the host cytoplasm and is capable of carrying out translation from the host ribosomal structure. Translation is a process by which viruses or other organisms can reproduce proteins. Direct ribosomal translation is a unique characteristic of positive sense RNA viruses. These RNA viruses also carry out RNA transcription in the host cell using RNA dependent RNA polymerase to create negative sense mRNA using positive sense mRNA as a template. This process repeats itself except the second time around negative sense mRNA, created the first time is used as a template to create positive mRNA. Through this process positive sense RNA viruses are more easily replicated than negative sense RNA viruses, which contributes to the degree of infectivity and disease prevalence associated with these viruses.

There are two discrete lineages of POWV that have been found in western hemisphere and Russia. Lineage I is the POWV and lineage II is the DTV. Despite some phylogenetic differences, the two lineages share 84% of the nucleotide and 94% of the amino acid sequence (23, 24). They also share antigenic similarity and are believed to have a common origin (25) from where they diverged 485 years ago (8). Serologically the two lineages cannot be differentiated and represent the two genotypes of the same virus (23, 24). The POWV lineage involves *I. cookei* and *Ixodes marxi* and mammals, like red squirrels, *Tamiasciurus hudsonicus*, ground hogs, *Marmota monax*, and skunks, *Mephitis* sp., while the DTV lineage involves *I. scapularis* (6). Even though Powassan is a *Flavivirus* consisting of miniature units around 50 nm (19), it exhibits great variability and genetic complexity. Based on detailed study of the genetic structure and complete decoding of the nucleotides, Powassan is the most diverse member of the *Flavivirus* family (26). POWV strain LB, was identified in human brain in Ontario, Canada in 1958 (26). POWV strain LEIV-3070Prm was segregated from *H. logicornis* (bush tick) in Primorsky Krai, Russia in 1977 (27). POWV LEIV-3070Prm shares 99.7% of similarity with POWV LB found in Canada and resembles 99.5% of the Powassan strains found in Far-Eastern Russia (27).

## Inoculation

Tick attachment to the host happens after successful “questing” (12). Compared to other tick-borne diseases, Powassan has a much shorter transmission time, as infection can occur 15 min after tick attachment (28). During the attachment, tick saliva serves as a local anesthetic to the skin and facilitates viral transmission (29). At the time of viral inoculation, the skin immune response changes gene expression to upregulate inflammatory cells (30). At 3 h post inoculation (hpi), inflammatory cytokines and chemokines like interleukin (IL)1B, IL6 IL 6A, toll-like receptor 4, and chemokine receptor type 3 (CCR3) are all upregulated (30). These inflammatory mediators in turn increase the number of phagocytes and neutrophils as part of the immune response (30). When the post inoculation response of POWV was studied up to 24 hpi, some interesting new findings were seen. It was found that neutrophils and mononuclear cells were still the primary leukocytes at the site of inoculation at 3 hpi; however, at 12 and 24 hpi, the immune response was much less robust as compared to 3 hpi (29). A unique finding was the presence of macrophages and fibroblast containing Powassan antigen at 12–24 hpi (29). Macrophages are phagocytes that engulf foreign antigen as well as cell debris, while fibroblast produce connective tissue and is a marker of tissue “repair.” The presence of Powassan antigen in these cells clearly shows that these cells were the target of infection (29). Downregulation of inflammatory cells at 12 to 24 hpi is also a clear indicator of tick saliva serving as an immune-modulator.

It has also been reported in a study by Hermance and Thangamani that mice that were inoculated with POWV in absence of tick saliva were able to survive the disease in contrast to those mice that were injected with the virus in presence of tick saliva (31). The presence of interleukins and other leukocytes at the site of viral insertion should be a deterrent to any pathogen in an immunocompetent individual. The progression of Powassan infection is an indication that tick saliva is not only shielding the virus but also downregulating the immune response at the local site of insertion (29). This downregulation is secondary to the multifaceted immunomodulation of tick saliva. However, it is unclear whether the immune-downregulation by tick saliva is due to “pharmacologically active components” in saliva (31) or POWV itself plays a significant role in immunosuppression at the cutaneous level. Once the virus penetrates the dermal and muscular layers and finds its way to the serum, it is only a matter of 8–34 days (32), in most cases, before the wide array of symptoms begin.

## Presentation and Symptoms

Powassan can be a rapidly progressing, neurological disease. Bacterial meningoencephalitis is characterized by a rapid invasion of the central nervous system (CNS), with signs of meningeal irritation and neurological deficits developing over the course of hours to days. However, tick-borne viral infections follow a more insidious course. Once inside the blood–brain barrier (BBB) and completely disseminated into the cerebrospinal fluid (CSF) and parenchymal tissue, tick-borne and non-tick-borne viral infections show a similar range of severity of symptoms. The symptoms of Powassan infection vary from person to person, as some are asymptomatic and others have a more progressive course.

**Figure 4:**
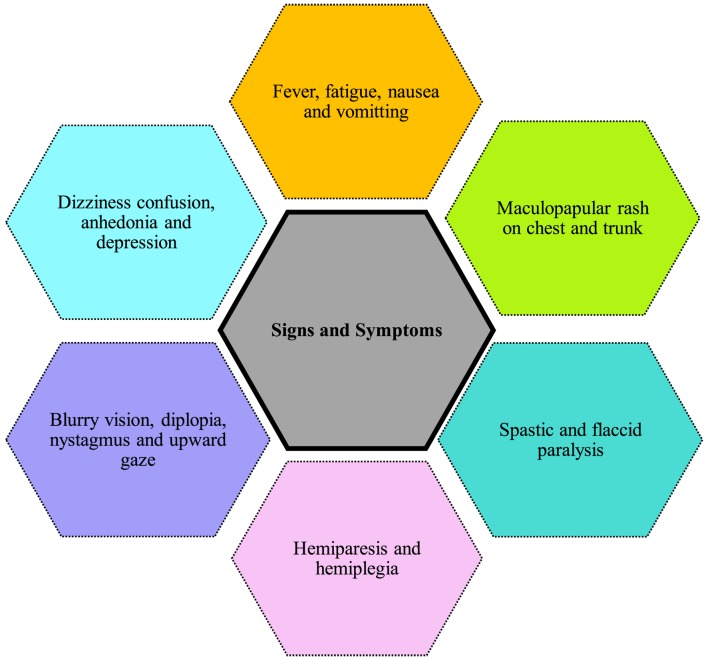
Signs and symptoms of Powassan virus; Ref. (32, 34, 35).

Figure 4 shows the symptoms reported in cases of Powassan infection. They include headache, fever, focal neurological deficits, confusion, generalized weakness, ataxia, somnolence, speech problems (aphasia, dysarthria), and seizures (33). The most common initial symptoms are headache and fever (34, 35). Although not present in majority of cases, a maculopapular rash on trunk and chest has been reported in few cases with no specific pathognomonic features (34). Powassan infection starts by mimicking a common cold, but rapidly advances into more severe symptoms. Neurological symptoms develop very rapidly after infection. Involvement of upper and lower motor neurons is common, and is more pronounced than that usually seen in other viral encephalitis. Other symptoms reported include spastic and flaccid paralysis (32), dizziness, nausea, vomiting, positive Babinski sign, blurry vision, diplopia, nystagmus, upward gaze palsy, and neuropsychiatric symptoms including anhedonia and depression (34, 35). Some cases also show intra-parenchymal hemorrhage and subdural hematoma causing focal neurological deficits like hemiplegia and hemiparesis. These patients lacked previous vascular pathology or bleeding diathesis, raising concerns Powassan may have actions similar to other hemorrhagic viruses like dengue and yellow fever.

Other *Falviviruses* like TBEV, West Nile virus (WNV), Japanese encephalitis virus (JEV), and St. Louis encephalitis virus (SLEV) also penetrate the BBB and cause neurologic symptoms, although their mode of entry into the brain varies. WNV and JEV gain entry into the CNS through infecting microvascular endothelial cells while SLEV enters the CNS through the olfactory neural pathway (36–38). TBEV has been noted to damage the endothelial lining of the vascular cells without breeching the BBB (39). The mechanism with which TBEV crosses the BBB is still unknown but study has shown that it infects the endothelial cells of the microvasculature and uses a cellular uptake mechanism to gain entry through the BBB (39). The exact mechanism of invasion for various neurotropic viruses is still an ongoing research and will require more in-depth analysis.

To gain understanding of the effects of POWV on various body tissues, the study by Santos et al. infected C57B6 mice with the virus (32). Tissues samples from different organs were collected, developed, and examined histologically. The three most commonly affected organs were the CNS, lymphoid tissue, and the spleen (32). In brain, both parenchymal and perivascular tissues were infiltrated by mononuclear cells with increase presentation of several cell turnover markers [Glial fibrillary acidic protein (GFAP) and CD11B]. GFAP is a marker for astrocytes while CD11b is a marker for microglial cells. Neurons throughout the CNS were commonly affected, as well as the anterior motor horn of the spinal cord. Outside the CNS, lymphoid tissue and spleen showed an increase in tangible body macrophages and white pulp hyperplasia. Viral antigen was recovered from cortical and pericortical sections of the spleen. Some perivascular involvement was also seen in the liver (32).

All of C57BL/6 mice that were inoculated with POWV in this study died (32). This is in spite of the fact that C57BL/6 mice are known to have some resistance to POWV (17). Necropsy of the mice with tissue analysis showed increased expression of the astrocyte marker, GFAP, and the macrophage (microglial) marker, CD11b. This rise in GFAP and CD11b was secondary to the invasion of Powassan into parenchymal tissue. Post infectious inflammatory and repair response from the cells led to the increase cell marker expression.

The study also showed changes to motor neurons in the ventral horn of the spinal cord. These are also the neurons affected by poliomyelitis. Flaccid paralysis, which represents a lower motor neuron lesion was observed in the mice that were inoculated with POWV. Both upper and lower motor neuron signs have been described in regard to Powassan cases (40–43). Upper motor neuron signs, like spastic paralysis and focal neurological deficits are an obvious presentation with parenchymal invasion and increased cell marker expression. However, the affinity of POWV for the ventral horn of spinal cord causing lower motor neuron sign is a unique feature that needs further study. It is a major concern to see a tick-borne infection show similar pathology to poliomyelitis.

There needs to be further evaluation of the perivascular infiltration in the CNS and liver. As stated previously, Powassan is known to have caused vascular insults and perivascular invasion by mononuclear infiltrates. Signs of bleeding in the CNS with no previous vascular anomalies could represent immune-complexes causing damage to the endothelial cell lining of CNS vessels. Further study is also needed to determine whether the intracerebral bleeding and perivascular deposition is secondary to the virus crossing the BBB or whether it is primarily an immune mediated mechanism. Immune mediators like cytokines, chemokines, and metalloproteinases play a significant role in compromising the BBB (44). Santos et al. (32) demonstrated an increased GFAP signal as a marker for astrocytes. GFAP is known to cause an increase in levels of MMP 9, which damages the BBB and is a mechanism by which *Flavivirus* have been known to invade the CNS (45). Overall the immune mediated process not only supports the breech of the BBB but also shows involvement in the vascular insult. There was also involvement of the spleen and the lymphoid system. Although there was no marrow invasion, the structural integrity of red blood cells could have been compromised due to splenic involvement, raising the concern for some kind of hematologic relationship behind the vascular insults.

## Diagnostic Challenges

### Imaging Studies

The diagnosis of tick-borne diseases represents a clinical challenge on multiple levels, as most disorders are diagnosed on a combination of clinical suspicion with serological and imaging evidence. Even though body fluids and tissues can be obtained for culture and further testing, there is still the uncertainty of the presence of an organism in that specific location. Infectious diseases are mercurial and diagnosis is not as straight forward as a biopsy that would yield in a neoplastic process. In Powassan infected patients with neurological symptoms, the work up starts with imaging in most cases, to rule out a mass lesion or cerebral hemorrhage as a cause of symptoms. Computed tomography (CT) scans and magnetic resonance imaging (MRI) of the brain are the two modes of imaging that are employed. CT scans are primarily X-ray-based imaging modality while MRI scans employ strong Tesla magnets to scan through body tissues. CT scans are very sensitive for blood and bone abnormalities as well as providing a good overview of any possible mass lesions with iodine based contrast agent being an enhancing option. MRI is an excellent tool for soft tissue detail and is primarily employed to pick up ischemia, in-depth analysis of mass lesions with or without gandolium based contrast agents as an enhancing option. The quality of the image is machine and operator dependent in most cases; however, MRI cannot be employed in emergency setting as it requires the patient to be still throughout the scan.

Acute presentations of Powassan will show no abnormalities on a CT scan unless there is intra-parenchymal bleeding and subdural hematoma (35). In cases where intracranial bleeding is seen, a further modified type of CT scan referred to as CT angiogram is utilized to visualize the vascular structures (35). CT angiogram in those cases showed no loss of vascular integrity. On MRI, some cases show no abnormalities or vascular insults, while others showed T2 fluid attenuated inversion recovery (FLAIR) hyperintensity (34). Hyper-intensities seen on T2 FLAIR sequence are a common imaging finding in the elderly and can be seen in variety of pathologies including ischemia, diseases of demyelination, and neuropsychiatric disorders. These hyper-intensities are most commonly seen in periventricular white matter, perivascular space and deep white matter. The presence of hyperintensity on MRI without other tissue abnormality suggests that MRI may be more useful than CAT scan as it relates to Powassan. The vascular insult and the T2 hyperintensity do improve with the resolution of symptoms (34, 35). The hyperintensity on the MRI is a marker of Powassan-related tissue or vascular changes as they disappear with symptomatic improvement while they are persisting when found incidentally in an elderly patient.

In cases from the Midwest it has been reported that the initial CT scan of the brain was unremarkable while the MRI showed non-specific hyperintensity in cases from the Northeast. The relatively small sample size (*n* = 4) from the Midwest study and the coincidental physician preference for MRI in Northeast and CT scan in the Midwest should be taken into consideration. However, these studies still raise a concern about the nature of Powassan that is present on the East Coast vs. the Midwest. *I. scapularis* is the main vector for transmitting Powassan on the East Coast, while *I. cookei* is the main vector in the Midwest. The question arises, does the vector also play a role in the way Powassan presents? Or does the vector somehow alter the virus?

### Serologic Testing and CSF Analysis

After imaging and clinical suspicion, serological data are obtained to support the diagnosis. CSF analysis and serum testing through different means is a key in establishing the diagnosis of any infectious process (Table 2). Tests like C-reactive protein, sedimentation rate, and complete blood count are too non-specific to establish the diagnosis in any infectious process. CSF analysis is crucial in conditions that involve the CNS, as neurological deficits are a clear indicator of breach of the BBB. In patients infected with Powassan, there was a prominent rise in protein and a variable rise in glucose, which was gradual in nature (34, 35). There was also leukocytosis with lymphocytic pleocytosis with a rise in nucleated segments in few instances (34, 35). Bacterial meningitis and encephalitis almost always give rise to neutrophils while viral processes cause an increase in lymphocytic count. However, under rare circumstances there is a rise in nucleated segments that is not typical for viral presentation and further complicates the pathophysiology of the disease.

**Table 2:** Cerebrospinal fluid analysis; Ref. (34, 46).

Condition	WBC (×10^6^/L)	Glucose (% of serum glucose)	Protein (g/dL)	Opening pressure (mmH_2_O)	Appearance
Normal	0–4	>60	<0.45	50–250	Clear
Powassan	Elevated	Mildly elevated	Elevated	–	–
Bacterial	1,000–5,000 polymorphs	Decreased	Increased	nl/Increased	Cloudy
Viral	10–2,000 lymphocytes	Normal	nl/Increased	Normal	Clear
SAH	nl/Slightly raised	Normal	Elevated	Elevated	Blood-stained xanthochromic
Tuberculosis	50–5,000 lymphocytes	Decreased	Elevated	nl/Elevated	Clear/cloudy

*SAH, subarachnoid hemorrhage; nl, normal*.

In addition to the cell count, CSF should also be examined using various methods to look for IgM antibody against POWV (34, 35). Elevation of IgM represents an acute or an active infection in most cases, while elevation of IgG only indicates the presence of antibody without any specification on it being an acute or a resolved infection. Enzyme-linked immunosorbent assay (ELISA) can be utilized to detect IgM antibodies in the CSF, although positive results are found only in a minority of cases (34). Even though CSF is the first place to show a non-specific change (the rise in cell count), a positive IgM will not be detected in CSF in the majority of cases. ELISA-based IgM and immunofluorescence antibody (IFA) assay should be done on a serum sample to check for active Powassan infection (47). Positive ELISA IgM and IFA can be confirmed by a plaque reduction neutralization test, which can enumerate the actual antibody titers (47). As compared to imaging, CSF analysis, and antibody testing, serum IgM testing is the most specific (34, 35). The lack of widely available serological tools is a major cause of delayed diagnosis of Powassan. Powassan testing is usually done only by state health departments and confirmed by the Center for Diseases Control and Prevention, as not all laboratories are equipped to carry out ELISA based IgM testing for Powassan (35). Because most Powassan cases present with non-specific symptoms, there is usually extensive testing for other tick-borne diseases before the possibility of Powassan is even considered (35). Even though the incidence of Powassan is rising, the comparative prevalence of Powassan compared to other tick-borne conditions is still quite low.

Another concern with tick-borne diseases is the possibility of concurrent infections. Most *flavivirus* are transmitted by a vector, either a mosquito or tick. The issue of concurrent infection is important not only due to the common vectors and geographic proximity but also due to the vague and non-specific presentation of these diseases. Frost et al. examined the serologic evidence of Lyme and Powassan as well as serologic evidence of Powassan with symptomatic IgM positive Lyme cases with consideration for cross reactivity that might exist due to similar antigenic properties (47). Overall 43.2% patients with some serologic evidence of Lyme diseases also had 17.1% serologic evidence of POWV. Lyme IgM was also detected in 85.7% of patients with evidence of sero-positivity for acute Powassan infection (47).

## Incidence and Prevalence

There were only a few cases of Powassan infection reported each year in the last half of the twentieth century in Canada and US, although Powassan was found in eastern Russia as well (6). However, the number of cases has increased considerably in the recent years in Canada and US, with reports of virus being found in Siberia, Alaska, and New Mexico (25). From 1958 to 1998, only 27 cases of POWV were detected throughout the world (3). However, there has been a sudden increase in the number of cases of POWV worldwide, especially in the US (3). From 1999 to 2016, 98 cases of Powassan infection were reported. The average cases/per year were 0.7 cases from 1958 to 1998 and the average rose to 5.4 cases/per year from 1999 to 2016 (3, 4). While there is a major lack of aggregated data and not all cases are reported, there has been at least a 671% rise in number of reported cases in the last 18 years compared to the 40 years before that. Emergence of a rare disease can be related not only to the vector and the antigenic drift and shift of the virus but also the host adaptation for new niche and habitat with concurrent changes in topography and climate. Previously the virus primarily infected a species of tick that did not bite humans. However, there has been a change in the ecology of Powassan, and now the more common deer tick has become infected (48). Since deer ticks do bite humans and are present in a wide geographic distribution, this has led to a rapid increase in the spread of POWV.

There are two major considerations that determine the geographic distribution of tick-borne diseases—the range of tick vectors and the spread of the virus within the vector population. Both Lyme and Powassan have shown “northward spread” from endemic areas in the US (49). The overall transmission of Powassan involves hosts like *Myodes* spp. voles in north, *Peromyscus* mice in south, *P*. *maniculatus* and *P*. *truei* in Mexico, and *M*. *rutilus* in Siberia (25). Since *I. scapularis* is a shared vector between Powassan and Lyme, the increasing prevalence of Lyme in Canada also raises concerns about emergence of babesiosis and granulocytic anaplasmosis (49).

Although Powassan is one of the least studied flaviviruses, there are few hypotheses on the spread of this disease. A study by Deardorff et al. discuss two hypotheses regarding geographic expansion of POWV (25). The first hypothesis suggests that tick-borne encephalitis Flaviviruses in the Old and New Worlds settled during the ice age in the “Palearctic and Nearctic” refuge areas and then expanded along the continental mainlands. The second hypothesis suggests that during the twentieth century, POWV was habituated from North America to Russia (25). Migration of mammals and moving warmer temperatures circulated the virus, consequently increasing its incidence. Neither of these hypotheses is independent of the other and the spread of POWV into the western hemisphere is still unclear. Other factors suggestive of Powassan spread are the increased population of white-tailed deer and migratory birds, which spread and introduce ticks to new regions (50).

While the origin of POWV is unknown, there is clear evidence that the spread of Powassan and other tick-borne diseases is highly dependent on the presence of ticks and their existence. Ticks live primarily in wooded or grassy areas, but are found even in urban woods and gardens. Tick survival is dependent on temperature, humidity, habitats, and the presence of other (10) “well-qualified requirements such as suitable host availability, temperature fluctuations between −10 and +35°C, and constant relative humidity not lower than 80% in the air” (50). Ticks thrive in a warm environment, which explains their population increase being directly proportional to the rise in temperature as a consequence of global warming. 2015 was the warmest year on record worldwide (51) and is a testament to the expected consequences of global warming. According to the research by Levi et al, 2050s would be much warmer compared to the first decade of 2000, which would advance nymph and larval phenology 10 days before the cycle time (52). The warming temperatures would be expected to increase the pathogenic transmission with advancing nymph and larval activity (52). There are also evidences suggesting that an increase in temperature will give rise to an increase in “overwinter survival, abundance, and geographical ranges of *Ixodid* tick vectors” (52). Statistically, from the 1900s, the global average surface temperature has increased at an average of 0.15°F per decade (51). Whether longer and warmer summers, shorter winters, or arctic ice plates melting, all these factors contribute to the increase in temperature, hence an increase in tick survival.

**Figure 5:**
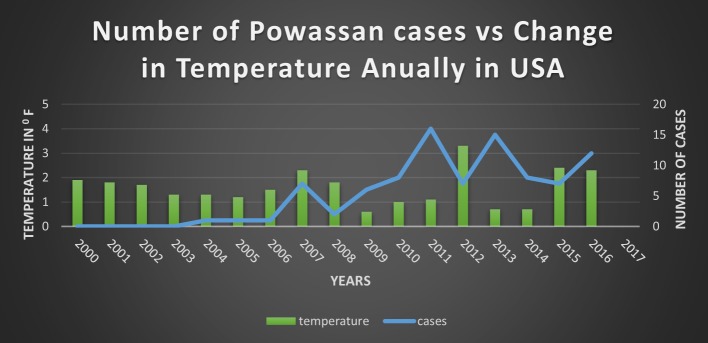
Comparison of temperature changes vs. diseases case, Ref. (4, 51).

Since 1895 there has been a significant change in annual temperatures of US with an increase ranging from 1.3–1.9°F (53–61) (Figure 5). Expansion of a diverse group of tick species would be predicted when there are warmer winters, and prolonged autumn and spring seasons (62). There are documented reports of expansion of tick due to the warmer weathers into the areas where they were not found before (50). Although warm weather is an important driving force for a wide expansion of the tick population, “changing rainfall patterns” might be playing a role as well (50).

Man-made deforestation, due to farming and logging operations contributes to global warming (63) and the resultant increase in the tick population. It has been estimated that deforestation is responsible for about 10% of total global warming emissions (63). Another factor contributing to the tick survival is humidity. Black legged nymph tick survival was decreased with exposure to dry air (64). Lower humidity is also fatal for hybrid ticks (65).

Tick density has been increasing annually in both southern and northern hemispheres, having a direct relationship to mild temperatures, low precipitation, low forest cover, and high urbanization (66). A study that collected ticks from 44 locations over a 7-year period showed that climate change and land use brought about changes in the density of *I. scapularis*. The change is the increased density of the tick which directly correlates to the increased incidence in tick-borne diseases (66).

Apart from ecological conditions, there are certain physiological features, which greatly affect tick survival and questing. Heat shock proteins (hsp) and stress response proteins (srp) are proteins that help protect organisms from damaging conditions like high temperature and humidity (50). Glutathione S-transferase, selenoproteins, metallothioneins, and ferritin are such proteins present in ticks which are involved in cellular responses to environmental stresses like heat shock, oxidative stress, tick attachment, blood feeding, and pathogen infection. Recent studies have shown that during exposure to warm temperatures and blood feeding, proteins like hsp20, hsp70, and subolesin are strongly activated in *I. scapularis*. During the process of blood feeding, tick develop immune response against pathogens attributing to their survival. These stress proteins not only help *I. scapularis* speed up the questing process but also protect them from environmental stress and pathogen infections.

As these ectoparasites expand worldwide, they also pose a threat to animal life (10) and destabilize vector and host equilibrium in animal kingdom. Ticks bring harm to animals in other ways than just transmitting diseases. Ticks, being the most important parasite that lives on cattle and other herbivores such as deer and mouse, lower the value of leather and cause anemia by sucking blood, which affects their growth, fertility, body weight, and milk production (10). There has been only limited study on Powassan infection in animals. Powassan has been reported to cause viremia with neurological symptoms in snowshoe hares (*Lepus americanus*) in Alberta, Canada (67) and wild murine rodents (68) and horses (10). Other Flaviviruses like TBEV, which cause human diseases in far-Eastern and Western Europe, also causes neurological damage in animals like, monkeys, horses, and dogs. Infected livestock animals like goats, sheep, and cows are known to transfer the virus through their milk onto their offspring and humans (10). Lyme disease also cause joint diseases in animals, and symptoms like loss of weight and appetite and renal diseases in dogs, cattle, horses, and cats (10). Thus, animals may also be subject to Powassan infection.

## Prevention

Tick-borne diseases all cause relatively non-specific, albeit sometimes very serious symptoms and diagnostic challenges, which make prevention of infection a priority. With the urbanization and climate change favoring growth in the tick populations, vector control is not the easiest of achievable goals. However, education on preventing tick bites can go a long way in preventing tick-borne diseases. Use of repellents and wearing long, light clothing when in wooded or grassy areas is important in decreasing tick contact. Knowledge of the signs of tick bites is also important. For tick-borne viral diseases, immunization is a crucial ingredient in developing host immune response. For the past 80 years, yellow fever live attenuated vaccine has substantially decreased the number of disease cases (18). Many cases of JEV have been prevented by live attenuated vaccines. However, at present there are no affective vaccination modalities available for zika virus, dengue or Powassan; although, efforts on creating and improving vaccination are under way (18). In general, live attenuated vaccines are known to cause competent immune response with the slight risk of active infection as a side effect. Live vaccines, for the most part, are given to immunocompetent individuals. With the rise in Powassan cases, development of a vaccine and post exposure prophylaxis protocol is an important public health priority.

Recently studies by Gomes-Solecki et al. and Tsao et al. suggest a pivotal role of outer surface protein A (OspA)-based vaccination in wildlife, which may reduce tick-borne pathogens in ticks (69, 70). OspA is an outer surface protein gene that encodes the outer membrane and is the main antigen of *Borrelia burgdorferi*, a predominant, causative agent of Lyme disease. The research showed how the Lyme infection in ticks was reduced when the ticks were fed on OspA-immunized mice. Even though Lyme is a bacterial infection, the idea of an outer surface protein based vaccination for wildlife spreading POWV can be as essential to the public health as was the vaccination for Lyme due to its good turnover. Regardless of the fact that the pathogen is viral, bacterial, or protozoal, by vaccinating target hosts and the use of pesticides would not only reduce the tick population but also decrease the pathogen transmission to humans and possibly decrease incidence of tick-borne diseases.

## Conclusion

Cases presenting to medical authorities, with neurological symptoms of unknown origin in a tick infested area, should always have Powassan encephalitis as a differential diagnosis. Most cases presenting with neurological deficits with non-specific signs indicating an infectious process are tested for various tiboviruses and other bacterial and protozoal infectious diseases before being tested for POWV. Even though the incidence of Powassan is on the rise, the lack of widely available testing and non-established protocols has played a significant part in delayed diagnosis. With the neurovirulent nature of the virus, any delays in diagnosis can leave long-lasting neurological sequels and in fact 50% of people with Powassan CNS involvement end up having long-lasting neurological insufficiencies. Serologic methods utilizing antibody testing hold great potential for achieving the diagnostic yield. Imaging findings are sensitive in ruling out other pathologies and can play a significant role in future research in establishing pathognomonic findings. However, they still lack the specificity to achieve the diagnostic yield. The most obvious diagnostic challenge is the lack of established guidelines. There is a great need to develop a testing protocol encompassing serologic methods, aspirational or biopsy techniques under the guidance of imaging or a combination of both. While standard guidelines are vital in any infectious process, equal emphasis should be placed on widely available serologic testing, which would substantially decrease the diagnostic turnaround time.

Tiboviruses not only cause significant damage by the types of disease they cause, in the context of humans and cattle, but also cause a significant fiscal strain on the health industry as extensive testing is required before the diagnostic yield is achieved. There should be strict emphasis on creating awareness about strategies on tick-bite prevention. There is also a need for vaccination development, post exposure prophylactic use of antivirals and immunoglobulins, and guidelines for treatment modalities should be established. Patients infected with Powassan are only given symptomatic and supportive treatment as there is no antiviral therapy that is FDA approved for Powassan treatment. With Powassan being one of the least studied flavivirus, despite its neurological severity and increase incidence, patients infected are tested and treated for other medical conditions ever before receiving any kind of supportive treatment for Powassan.

## Author Contributions

SF is the first author and RZ is the co-author; they did the review and created the first drafts and DC provided the overall guidance and direction to the project.

## Conflict of Interest Statement

The authors declare that the research was conducted in the absence of any commercial or financial relationships that could be construed as a potential conflict of interest.

## References

[1] Kazimírová M Thangamani S Bartíková P Hermance M Holíková V Štibrániová I Tick-borne viruses and biological processes at the tick-host-virus interface. Front Cell Infect Microbiol (2017) 7:339. 10.3389/fcimb.2017.00339 28798904 PMC5526847

[2] Hermance ME Thangamani S. Powassan virus: an emerging arbovirus of public health concern in North America. Vector Borne Zoonotic Dis (2017) 17(7):453–62. 10.1089/vbz.2017.2110 28498740 PMC5512300

[3] Hinten SR Beckett GA Gensheimer KF Pritchard E Courtney TM Sears SD Increased recognition of Powassan encephalitis in the United States, 1999–2005 vector-borne and zoonotic diseases. Vector Borne Zoonotic Dis (2008) 8(6):733–40. 10.1089/vbz.2008.0022 18959500

[4] U.S. Geological Survey Disease Maps. Powassan Virus (2016). Available from: http://diseasemaps.usgs.gov/mapviewer

[5] McLean DM McQueen EJ Petite HE MacPherson LW Scholten TH Ronald K Powassan virus: field investigations in Northern Ontario, 1959 to 1961. Can Med Assoc J (1962) 86(21):971–4. 20327133 PMC1849058

[6] Anderson JF Armstrong PM. Prevalence and genetic characterization of Powassan virus strains infecting *Ixodes scapularis* in Connecticut. Am J Trop Med Hyg (2012) 87(4):754–9. 10.4269/ajtmh.2012.12-0294 22890037 PMC3516331

[7] Center for Disease Control and Prevention. Tickborne Diseases of the United States (2017). Available from: https://www.cdc.gov/ticks/diseases/index.html

[8] Pesko KN Torres-Perez F Hjelle BL Ebel GD. Molecular epidemiology of Powassan virus in North America. J Gen Virol (2010) 91(Pt 11):2698–705. 10.1099/vir.0.024232-0 20631087 PMC3052558

[9] Companion Vector Borne Diseases. General Aspects, Taxonomy (1991). Available from: http://www.cvbd.org/en/tick-borne-diseases/about-ticks/general-aspects/taxonomy/

[10] Brites-Neto J Duarte KMR Martins TF. Tick-borne infections in human and animal population worldwide. Vet World (2015) 8(3):301–15. 10.14202/vetworld.2015.301-315 27047089 PMC4774835

[11] Guglielmone AA Robbins RG Apanaskevich DA Petney TN Estrada-Pena A Horak IG The argasidae, ixodidae and nuttalliellidae (Acari: Ixodida) of the world list of valid species names. Exp Appl Acarol (2002) 28:27–54. 10.1023/A:1025381712339 14570115

[12] Center for Disease Control and Prevention. Lifecycle of Hard Ticks That Spread Disease. Available from: https://www.cdc.gov/ticks/life_cycle_and_hosts.html

[13] Center for Disease Control and Prevention. DPDx-Laboratory Identification of Parasitic Diseases of Public Health Concern (2016). Available from: https://www.cdc.gov/dpdx/ticks/index.html

[14] Center for Disease Control and Prevention. Tick Borne Diseases of the USA-A Reference Manual for Health Care Professionals (2007). Available from: https://www.cdc.gov/lyme/resources/tickbornediseases.pdf

[15] The University of Maine. Ticks-Ixodes cookei (2016). Available from: https://extension.umaine.edu/home-and-garden-ipm/frequent-specimens/frequent-ticks/ixodes-cookei-ticks-2/.

[16] The University of Maine. Tick Species of Maine (2008). Available from: https://extension.umaine.edu/ipm/tickid/maine-tick-species/woodchuck-tick/.

[17] Mlera L Meade-White K Saturday G Scott D Bloom ME. Modeling Powassan virus infection in *Peromyscus leucopus*, a natural host. PLoS Negl Trop Dis (2017) 11(1):e0005346. 10.1371/journal.pntd.0005346 28141800 PMC5302833

[18] Collins MH Metz SW. Progress and works in progress: update on flavivirus vaccine development. Clin Ther (2017) 39(8):1519–36. 10.1016/j.clinthera.2017.07.001 28754189

[19] P-Lindenbach BD Thiel H-J Rice CM Chapter 33: flaviviridae: the viruses and their replication. 5th ed In: Knipe DM Howley PM, editors. Fields Virology. New York, NY: Lippincott Williams & Wilkins (2006) 4–5.

[20] Huang Y-JS Higgs S Horne KM Vanlandingham DL. Flavivirus-mosquito interactions. Viruses (2014) 6(11):4703–30. 10.3390/v6114703 25421894 PMC4246245

[21] Centers for Disease Control and Prevention. Viral Hemorrhagic Fever (2014). Available from: https://www.cdc.gov/vhf/virus-families/flaviviridae.html

[22] Centers for Disease Control and Prevention. Dengue (2014). Available from: https://www.cdc.gov/dengue/epidemiology/index.html

[23] Beasley DW Suderman MT Holbrook MR Alan Barrett AD. Nucleotide sequencing and serological evidence that the recently recognized deer tick virus is a genotype of Powassan virus. Virus Res (2001) 79(1–2):81–9. 10.1016/S0168-1702(01)00330-6 11551648

[24] Kuno G Artsob H Karabatsos N Tsuchiya KR Chang GJ Genomic sequencing of deer tick virus and phylogeny of Powassan-related viruses of North America. Am J Trop Med Hyg (2001) 65(5):671–6. 10.4269/ajtmh.2001.65.671 11716135

[25] Deardorff ER Nofchissey RA Cook JA Hope AG Tsvetkova A Talbot SL Powassan virus in mammals, Alaska and New Mexico, USA, and Russia, 2004–2007. Emerg Infect Dis (2013) 19(12):2012–6. 10.3201/eid1912.130319 24274336 PMC3840874

[26] Mendl CW Holzmann H Kunz C Heinz FX. Complete genomic sequence of Powassan virus: evaluation of genetic elements in tick-borne versus mosquito-borne flaviviruses. Virology (1993) 194(1):173–84. 10.1006/viro.1993.1247 8097605

[27] Lvov DK Alkhovsky SV Shchelkanov MY Shchetinin AM Deryabin PG Gitelman AK Genetic characterisation of Powassan virus (POWV) isolated from *Haemaphysalis longicornis* ticks in Primorye and two strains of tick-borne encephalitis virus (TBEV) (Flaviviridae, Flavivirus): Alma-Arasan virus (AAV) isolated from *Ixodes persulcatus* ticks in Kazakhstan and Malyshevo virus isolated from *Aedes vexans* nipponii mosquitoes in Khabarovsk kray. Vopr Virusol (2014) 59(5):18–22. 25895206

[28] Ebel GD Kramer LD. Short report: duration of tick attachment required for transmission of Powassan virus by deer ticks. Am J Trop Med Hyg (2004) 71(3):268–71. 15381804

[29] Hermance ME Santos RI Kelly BC Valbuena G Thangamani S. Immune cell targets of infection at the tick-skin interface during Powassan virus transmission. PLoS One (2016) 11(5):e0155889. 10.1371/journal.pone.0155889 27203436 PMC4874601

[30] Hermance ME Thangamani S Proinflammatory cytokines and chemokines at the skin interface during Powassan virus transmission. J Invest Dermatol (2014) 134(8):2280–3. 10.1038/jid.2014.150 24658509 PMC4102615

[31] Hermance ME Thangamani S. Tick saliva enhances Powassan virus transmission to the host, influencing its dissemination and the course of disease. J Virol (2015) 89(15):7852–60. 10.1128/JVI.01056-15 25995246 PMC4505606

[32] Santos RI Hermance ME Gelman BB Thangamani S. Spinal cord ventral horns and lymphoid organ involvement in Powassan virus infection in a mouse model. Viruses (2016) 8(8):220. 10.3390/v8080220 27529273 PMC4997582

[33] Center for Disease Control and Prevention. Powassan Virus, Signs and Symptoms (2015). Available from: https://www.cdc.gov/powassan/symptoms.html

[34] Piantadosi A Rubin DB McQuillen DP Hsu L Lederer PA Ashbaugh CD Emerging cases of Powassan virus encephalitis in New England: clinical presentation, imaging, and review of the literature. Clin Infect Dis (2016) 62(6):707–13. 10.1093/cid/civ1005 26668338 PMC4850925

[35] Raval M Singhal M Guerrero D Alonto A. Powassan virus infection: case series and literature review from a single institution. BMC Res Notes (2012) 5:594. 10.1186/1756-0500-5-594 23111001 PMC3506459

[36] Verma S Lo Y Chapagain M Lum S Kumar M Gurjav U West Nile virus infection modulates human brain microvascular endothelial cells tight junction proteins and cell adhesion molecules: transmigration across the in vitro blood-brain barrier. Virology (2009) 385(2):425–33. 10.1016/j.virol.2008.11.047 19135695 PMC2684466

[37] Li F Wang Y Yu L Cao S Wang K Yuan J Viral infection of the central nervous system and neuroinflammation precede blood-brain barrier disruption during Japanese encephalitis virus infection. J Virol (2015) 89(10):5602–14. 10.1128/JVI.00143-15 25762733 PMC4442524

[38] Monath TP Cropp CB Harrison AK Mode of entry of a neurotropic arbovirus into the central nervous system. Reinvestigation of an old controversy. Lab Investig (1983) 48(4):399–410. 6300550

[39] Palus M Vancova M Simarova J Elsterova J Perner J Ruzek D. Tick-borne encephalitis virus infects human brain microvascular endothelial cells without compromising blood-brain barrier integrity. Virology (2017) 507:110–22. 10.1016/j.virol.2017.04.012 28432926

[40] Rossier E Harrison RJ Lemieux B A case of Powassan virus encephalitis. Can Med Assoc J (1974) 110(10):1173–80. 4829843 PMC1947498

[41] Wilson MS Wherrett BA Mahdy MS Powassan virus meningoencephalitis: a case report. Can Med Assoc J (1979) 121(3):320–3. 223757 PMC1704315

[42] Hicar MD Edwards K Bloch K. Powassan virus infection presenting as acute disseminated encephalomyelitis in Tennessee. Pediatr Infect Dis J (2011) 30(1):86–8. 10.1097/INF.0b013e3181f2f492 20736878

[43] Gholam BI Puksa S Provias JP Powassan encephalitis: a case report with neuropathology and literature review. CMAJ (1999) 161(11):1419–22. 10906899 PMC1230834

[44] Růžek D Salát J Singh SK Kopecký J. Breakdown of the blood-brain barrier during tick-borne encephalitis in mice is not dependent on CD8^+^ T-cells. PLoS One (2011) 6(5):e20472. 10.1371/journal.pone.0020472 21629771 PMC3100324

[45] Palus M Bily T Elsterova J Langhansova H Salat J Vancova M Infection and injury of human astrocytes by tick-borne encephalitis virus. J Gen Virol (2014) 95:2411–26. 10.1099/vir.0.068411-0 25000960

[46] Colledge NR Walker BR Ralston SH Davidson S Chapter 26: neurological disease. Davidson’s Principles & Practice of Medicine (International Edition). 21st ed Churchill Livingstone/Elsevier (2010) 1141–47.

[47] Frost HM Schotthoefer AM Thomm AM Dupuis AP Kehl SC Kramer L Serologic evidence of Powassan virus infection in patients with suspected Lyme disease. Emerg Infect Dis (2017) 23(8):1384–8. 10.3201/eid2308.161971 28726610 PMC5547799

[48] Yale School of Public Health. The Rise of Powassan Virus (2015). Available from: http://publichealth.yale.edu/news/archive/article.aspx?id=9147

[49] Kulkarni MA Berrang-Ford L Buck PA Drebot MA Lindsay LR Ogden NH Major emerging vector-borne zoonotic diseases of public health importance in Canada. Emerg Microbes Infect (2015) 4(6):e33 10.1038/emi.2015.33 26954882 PMC4773043

[50] Estrada-Peña A Ayllón N de la Fuente J. Impact of climate trends on tick-borne pathogen transmission. Front Physiol (2012) 3:64. 10.3389/fphys.2012.00064 22470348 PMC3313475

[51] United States Environmental Protection Agency. Climate Change Indicators: U.S. and Global Temperature (2016). Available from: https://www.epa.gov/climate-indicators/climate-change-indicators-us-and-global-temperature

[52] Levi T Keesing F Oggenfuss K Ostfeld RS. Accelerated phenology of blacklegged ticks under climate warming. Philos Trans R Soc Lond B Biol Sci (2015) 370(1665):556. 10.1098/rstb.2013.0556 25688016 PMC4342961

[53] Global Change. Recent US Temperature Trends (2014). Available from: http://nca2014.globalchange.gov/report/our-changing-climate/recent-us-temperature-trends#statement-16553

[54] Global Climate Change Impacts in the United States, U.S. Global Change Research Program, A State of Knowledge Report from the U.S. Global Change Research Program. Cambridge University Press (2009). Available from: https://www.climatecommunication.org/wp-content/uploads/2013/04/climate_impacts.pdf

[55] Fall S Niyogi D Gluhovsky A Pielke RA Kalnay E Rochon G Impacts of land use land cover on temperature trends over the continental United States: assessment using the North American Regional Reanalysis. Int J Climatol (2010) 30:1980–93. 10.1002/joc.1996

[56] Fall S Watts A Nielsen-Gammon J Jones E Niyogi D Christy JR Analysis of the impacts of station exposure on the U.S. Historical Climatology Network temperatures and temperature trends. J Geophys Res (2011) 116:D14120 10.1029/2010JD015146

[57] Menne MJ Williams CN Palecki MA On the reliability of the U.S. surface temperature record. J Geophys Res (2010) 115:D11108 10.1029/2009JD013094

[58] Menne MJ Williams CN Vose RS The U.S. Historical Climatology Network monthly temperature data, version 2. Bull Am Meteorol Soc (2009) 90:993–1007. 10.1175/2008BAMS2613.1

[59] Menne MJ Williams CN Homogenization of temperature series via pairwise comparisons. J Clim (2009) 22:1700–17. 10.1175/2008JCLI2263.1

[60] Vose RS Applequist S Menne MJ Williams CN Thorne P An intercomparison of temperature trends in the U.S. Historical Climatology Network and recent atmospheric reanalyses. Geophys Res Lett (2012) 39:L10703 10.1029/2012GL051387

[61] Williams CN Menne MJ Thorne PW Benchmarking the performance of pairwise homogenization of surface temperatures in the United States. J Geophys Res (2012) 117:D05116 10.1029/2011JD016761

[62] Dantas-Torres F. Climate change, biodiversity, ticks and tick-borne diseases: the butterfly effect. Int J Parasitol Parasites Wildl (2015) 4(3):452–61. 10.1016/j.ijppaw.2015.07.001 26835253 PMC4699983

[63] Union of Concerned Scientist. Tropical Deforestation and Global warming. Available from: http://www.ucsusa.org/global_warming/solutions/stop-deforestation/tropical-deforestation-and-1.html#.WYjlc7pFzmQ

[64] Rodgers SE Zolnik CP Mather TN. Duration of exposure to suboptimal atmospheric moisture affects nymphal blacklegged tick survival. J Med Entomol (2007) 44(2):372–5. 10.1093/jmedent/44.2.372 17427711

[65] United States Geological Survey. It’s the Heat and the Humidity, New Study Finds: Why Lyme Disease Is Common in the North, Rare in the South (2017). Available from: https://www.usgs.gov/news/it-s-heat-and-humidity-new-study-finds-why-lyme-disease-common-north-rare-south

[66] Khatchikian CE Prusinski M Stone M Backenson PB Wang I-N Levy MZ Geographical and environmental factors driving the increase in the Lyme disease vector *Ixodes scapularis*. Ecosphere (2012) 3(10):art85. 10.1890/ES12-00134.1 24371541 PMC3872055

[67] Zarnke RL Yuill TM Powassan virus infection in snowshoe hares (*Lepus americanus*). J Wildl Dis (1981) 17(2):303–10. 10.7589/0090-3558-17.2.303 6264168

[68] Leonova GN Krugliak SP Lozovskaia SA Rybachuk VN The role of wild murine rodents in the selection of different strains of tick-borne encephalitis and Powassan viruses. Vopr Virusol (1987) 32(5):591–5. 2829440

[69] Gomes-Solecki MJ Brisson DR Dattwyler RJ. Oral vaccine that breaks the transmission cycle of the Lyme disease spirochete can be delivered via bait. Vaccine (2006) 24(20):4440–9. 10.1016/j.vaccine.2005.08.089 16198456

[70] Tsao K Fish D Galvani AP. Predicted outcomes of vaccinating wildlife to reduce human risk of Lyme disease. Vector Borne Zoonotic Dis (2012) 12(7):544–51. 10.1089/vbz.2011.0731 22251312

